# Physiological response and cardiorespiratory adaptation after a 6-week Nordic Walking training targeted at lipid oxidation in a group of post-menopausal women

**DOI:** 10.1371/journal.pone.0230917

**Published:** 2020-04-01

**Authors:** Agata Cebula, Anna Katarzyna Tyka, Aleksander Tyka, Tomasz Pałka, Wanda Pilch, Lidia Luty, Dariusz Mucha

**Affiliations:** 1 Department of Biological Regeneration and Posture Correction, Faculty of Physical Education and Sport, University of Physical Education, Krakow, Poland; 2 Department of Recreation and Biological Regeneration, Faculty of Tourism and Leisure, University of Physical Education, Krakow, Poland; 3 Department of Physiology and Biochemistry, Faculty of Physical Education and Sport, University of Physical Education, Krakow, Poland; 4 Department of Biochemistry and Basic of Cosmetology, Faculty of Cosmetology, University of Physical Education, Krakow, Poland; 5 Department of Statistics and Econometrics, University of Agriculture, Krakow, Poland; Universidade Federal de Mato Grosso do Sul, BRAZIL

## Abstract

This study examined the effects of a 6-week Nordic Walking (NW) training, at the intensity corresponding to the dominance of lipid metabolism, on the levels of selected physiological indices, the haemodynamic indices of the cardiovascular system and physical fitness in sedentary women older than 55 years of age. In addition, the physiological response of the female body to the walking effort on treadmill with poles (NW) and without poles (W) was compared and the influence of training on this response was determined. A single group study with a pre-test/post-test study design was conducted. Eighteen women performed NW controlled intensity training 3 times a week for 6 weeks. Body composition, resting blood pressure (BP), heart rate (HR), maximum oxygen uptake (VO_2_max) as well as circulatory and respiratory indices in two graded walking efforts on mechanical treadmill NW and W were measured before and after training period. The intensity of workouts, which considered the dominance of lipid metabolism, was determined individually, based on the dynamics of changes in the level of physiological indices during the graded intensity NW. After the course of training, body mass, fat mass, resting BP and HR decreased significantly (p < 0.05). HR and respiratory exchange ratio recorded during NW and W at 1.75 m^.^s^-1^ walking speed decreased, while the oxygen pulse increased (p < 0.05). VO_2_max increased significantly (p < 0.05). Before and after the training period HR, oxygen uptake per minute, and energy expenditure during NW were significantly higher than in W (p < 0.05). The study showed that 6-week NW training at the intensity corresponding to the dominance of lipid metabolism can provide improvement in body composition, cardiovascular function and physical performance in previously sedentary women. NW compared to the regular walk with the same speed revealed higher energy expenditure.

## Introduction

Involutional changes occurring in an aging body increase disease incidence. Aging is an independent factor which increases the risk of arterial stiffness and hypertension, and cardiovascular aging is a recognised factor determining life expectancy [1]. Several functional changes and compensatory responses which take place in an aging heart reduce its ability to respond to an increased workload and reduce the functional reserve. This, in turn, increases the tendency of myocardial damage. The effects of aging on the cardiovascular system are reported much more frequently during exercise than at rest. The general decrease in exercise tolerance recorded with age is manifested i.a. by a progressive decrease in the value of maximum oxygen uptake (VO_2_max) by an average of 8–10% per decade, starting at the age of 20–30. In subsequent years it accelerates to 20–25% per decade [2]. It has been shown that a low level of cardiorespiratory fitness (VO_2_max), similarly to hypertension, is associated with an increased risk of cardiovascular disease (CVD) and a higher risk of all-cause mortality [3].

Regular physical activity, which should include endurance, strength and stretching exercises, plays a key role in the process of aging with grace [3]. Regular endurance effort may increase aerobic capacity in older people [4,5], improving their quality of life and prolonging their ability to live independently. In addition, aerobic exercises positively influence body composition, reduce inflammatory processes, regulate lipid metabolism and blood glucose levels [6,7].

In the category of endurance exercises, alongside ordinary walking (W), Nordic Walking (NW) is more and more frequently recommended for the elderly. The advantage of NW lies in the fact that with the right technique of walking with poles, the mobilization of the upper body is significantly greater compared to W, which, in turn, results in a higher intensity effort with a comparable rate of perceived exertion (RPE) [8–12].

Along with the volume and the weekly frequency of exercise in the health training of the elderly, the intensity of effort seems to play a significant role. In previous research examining the health-promoting effects of NW on the body, mainly the moderate intensity effort was predominant, which was determined in a standard manner, e.g. based on a specific percentage of heart rate reserve (%HRR), percentage of maximum heart rate (%HRmax) or percentage of maximal oxygen uptake (%VO_2_max) [6,13–16]. In some studies, the intensity of training was determined based on the subject’s perception of exertion (RPE-Rating of Perceived Exertion) and walking speed (e.g. ‘brisk walking,’ ‘preferred or maximum walking speed’) [17–20]. Although in all of these cases the intensity of the exercise seemed to correspond to the intensity recommended for the improvement of health [3], the choice of such intensity at NW training was not sufficiently justified. For instance, it is unclear which energy substrates were the source of energy for working muscles during the training. There is evidence that the endurance training with individualized intensity oriented on the maximum mobilisation of the body’s fat reserves (Fatmax, LIPOXmax) can quickly lead to the improvement of body composition, cardiovascular condition, lipid metabolism, and can result in lowering blood pressure [21–23]. Therefore, it seems likely that such intensity of exercise may be particularly beneficial in health training of the elderly [24]. However, the physiological response of the body and the adaptive cardiovascular response to such NW intervention have not been studied.

Accordingly, the main goal of the study was (1) to determine the influence of a 6-week NW training at the intensity aimed at lipid oxidation at the levels of selected physiological indices, haemodynamic indices of the cardiovascular system, and physical fitness in a group of post-menopausal women. An additional objective of the study was (2) to compare the body’s physiological response to walking effort on mechanical treadmill with poles (NW) and without poles (W), and to determine the effect of training on this response. The following hypotheses were tested: (1) Nordic walking training with an intensity level focused on the dominance of fat metabolism improves the level of various health-related physiological and haemodynamic indices in post-menopausal women. (2) Regardless of the training intervention, NW compared to W at the same speed generates higher energy expenditure.

## Materials and methods

### Study population

Subject were recruited in southern Poland through printed and electronic advertisements on notice boards located in public places. Advertising through printed media (local newspapers) was also used. Recruitment was carried out at the Institute of Biomedical Science in the University of Physical Education in Krakow from May to July 2013. Out of the 86 volunteers who agreed to participate, 18 women (aged 57.8 ± 2.01 years, height 161.2 ± 5.48 cm) met the eligibility criteria and were enrolled in the study. The inclusion criteria were as follows: female aged above 55, with no medical contraindications to stress tests, i.e. generally in good health (no diagnosed cardiovascular, pulmonary, neurological, nephrological, metabolic or oncological diseases), no musculoskeletal disorders that can limit exercise performance, who have undergone menopause (no menstruation for at least 12 months), and low level of physical activity determined according to the International Physical Activity Questionnaire [IPAQ]—Polish abridged version [25]. The study excluded individuals taking medication and supplements that can affect levels of the assessed indices, alcohol users, smokers, women on elimination or low-calorie diet within 6 months prior to the survey, and women who had regularly practiced walking with poles or had been doing some other form of endurance exercise. All the subjects came from the southern part of Poland.

Over the entire course of the study, the subjects were advised not to change their usual diet, refrain from drinking alcohol and practising additional forms of physical activity.

All the subjects gave their written consent to participate in the study and were informed about the possibility of quitting at any stage without giving a reason. The study was conducted pursuant to the Declaration of Helsinki (1964) and was approved by the Bioethics Committee of the District Medical Chamber in Krakow (No. 83/KBL/OIL/2011).

### Experimental procedures and protocols

**Fig 1:**
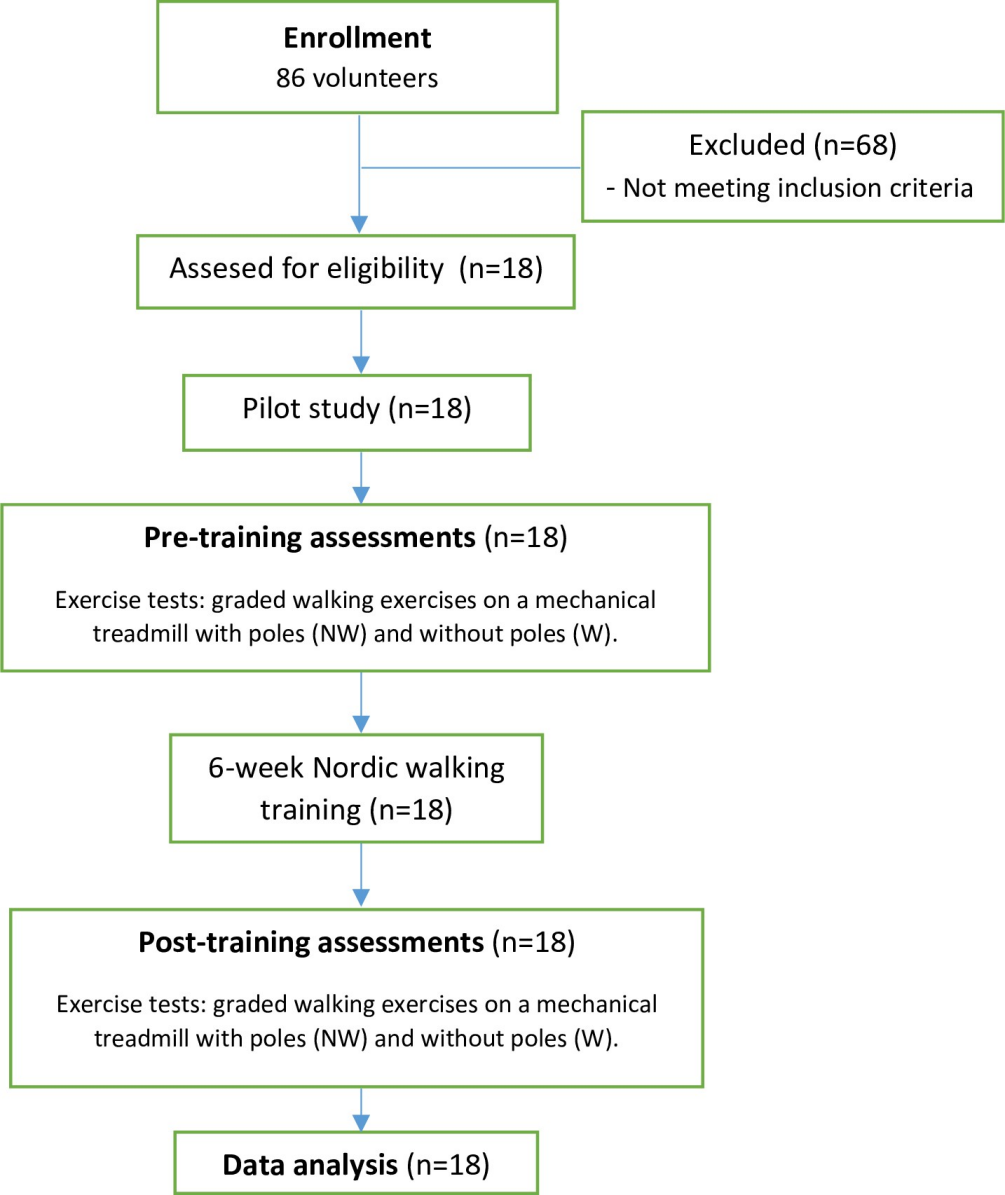
Flowchart of the study design.

In this study, quasi-experimental research methodology has been used in the framework of pre-test/post-test design. All evaluations and training sessions were conducted in the facilities of the University of Physical Education in Krakow (Poland). The familiarisation period took place in August 2013, and the pre-training data collection in September 2013. Training period (September/October) and post-training data collection (October/November) were performed in 2013. A pilot study was carried out first to familiarise the subjects with the testing procedure and the measuring equipment. The main study was conducted in two identical series. The first one took place a week before the start of the course of trainings, after the subjects mastered the technique of walking with poles; the second series took place on the second and the fourth day after the last training (Fig 1).

In each series of tests, during the first stage, anthropometric measurements and measurements of resting blood pressure were taken. In the first series of tests, women were randomly divided into two equal groups (Group I and Group II). Group I performed a graded walk on a mechanical treadmill without poles (W), and on the second day after W they performed an identical graded walk on the same mechanical treadmill, but this time with poles (NW). Group II performed the same graded walking exercise but in reverse order. The order of graded exercises was the same in the measurements after the training period as in the initial measurements (Fig 1).

Each of the graded exercises was conducted no earlier than 2 hours after a light meal. The levels of selected cardiorespiratory indices were recorded during the exercises and right after them.

In order to avoid the influence of diurnal variation at the level of the analysed variables, the graded exercise and NW training in the field were conducted in the morning, between 8.00 and 10.00 a.m.

### Training protocols and monitoring

The training was 6 weeks long and was performed outdoors, on a grassy compacted ground, under the supervision of a certified NW coach. The workouts were conducted no earlier than 2 hours after a light meal, 3 times a week for 90 minutes each, and included a 15-minute warm-up, a 60-minute main part during which women marched at individually determined intensity, and the final 15 minutes of relaxing and stretching exercises.

During the NW graded exercise the respiratory exchange ratio (RER) was established, which allowed to determine individual training intensity. The range of RER was selected based on the dominant percentage of use of free fatty acids (FFA). The intensity of the training exercise was determined by heart rate (HR) measured at the level of RER = 0.70±0.04 [26].

Telemetry monitoring of HR was used during the training. In the main part of each workout, the subjects were advised to maintain their HR within the given personal range (HRrange ± 4 beats^.^min^-1^), which was checked after each training based on HR record from the cardio-monitor. In three selected training sessions (weeks 1, 4 and 6) in the 30^th^ minute of the main part, the level of respiratory exchange rate (FEO_2_ –fraction of oxygen in expired air; VO2 –oxygen uptake per minute; RER–respiratory exchange ratio) was recorded using a portable ergospirometer (Start 2000M, MES, Poland), which enabled the control of training loads and their possible modification.

### Anthropometric measurements

Anthropometric measurements were taken in the morning after an overnight fasting, after bowel movement. The body height was measured with the Martin anthropometer (USA) with 1-mm accuracy. Body mass (BM), body fat percentage (PBF%), lean body mass (LBM) and fat mass (FM) were calculated by electrical bio-impedance method (IOI-353 Jawon Medical, Korea), following the guidelines to ensure accuracy [27]. Body Mass Index (BMI) was calculated by dividing the person’s weight (kg) by their height squared (m^2^).

### Haemodynamic measurements

Heart rate (HR) and blood pressure (BP) were measured in the morning after a 30-minute rest in a sitting position. HR was measured telemetrically with a Polar Team^2^ cardio-monitor (Polar Electro, Finland). Arterial BP was measured three times at the level of the brachial artery with a 2-minute interval, using a mercury manometer and the Korotkoff Sounds method, with a 5-mmHg (0.67 kPa) accuracy. Resting BP was calculated as the mean value of three consecutive measurements. The measurements were always taken by the same person.

### Measurements of physiological parameters

The level of respiratory exchange rates and cardiovascular indices were recorded during two identical graded walking exercises on a mechanical treadmill with poles (NW) and without poles (W). Both exercise tests were performed on h/p/cosmos Saturn 250/100R mechanical treadmill (h/p/cosmos, Germany). Each test began with a 3-minute warm-up at an initial walking speed of 1 m^.^s^-1^, after which speed was gradually increased by 0.25 m^.^s^-1^ every 3 minutes. The exercise test was carried out up to the fourth load segment, i.e. up to the 1.75 m^.^s^-1^ walking speed. During the graded exercise, the respiratory exchange rates were recorded every 30 seconds using the Start 2000M ergospirometer (MES, Poland). HR was measured using HR monitor (Polar Team^2^, Polar Electro, Finland) prior to, during, and 3 minutes after the exercise test. BP was measured in a sitting position in the third minute after completing the test, using the Korotkoff Sounds method.

The calculation of gross energy expenditure (EE) during exercise tests on a mechanical treadmill, in the segment at the walking speed of 1.75 m^.^s^-1^ in the ‘steady state,’ was performed using the simplified indirect calorimetry method, not including oxygen debt. The value of energy equivalent (calorific equivalent) for oxygen was determined according to the table suggested by Péronnet and Massicotte [26] based on non-protein values of respiratory exchange ratio (RER). Formula 1 was used [28]:

$$kcal=VO_{2}\times caloric\;equivalent\;for\;RER\times DE$$ (1)

where:

VO_2_ is oxygen uptake per minute [l^.^min^-1^],

Caloric equivalent for Respiratory Exchange Ratio [kcal^.^l^-1^],

DE is the duration of the exercise [min].

The sum of heart rate (∑HR) was calculated by adding HR values recorded in each full minute of graded effort until the end of the segment with 1.75 m^.^s^-1^ walking speed.

Heart rate recovery index (HRR%) was calculated according to formula 2 [29]:

$$HRR\%=\left[\left(HR_{2}‐HR_{3}\right)/\left(HR_{2}‐HR_{1}\right)\right]\times 100$$ (2)

where:

HR_1_ is heart rate before the exercise [beats^.^min^-1^],

HR_2_ is maximum heart rate (HR_max_) during the exercise [beats^.^min^-1^],

HR_3_ is heart rate in the 3^rd^ minute after the exercise [beats^.^min^-1^].

ΔHR_max-3ʼ_ was also calculated–the absolute difference between maximum heart rate recorded in the graded exercise and heart rate in the third minute after finishing the exercise.

### Maximal oxygen uptake

Maximum oxygen uptake (VO_2_max) was estimated using a sub-maximum walking test on a mechanical treadmill. Oxygen uptake per minute (VO_2_) and heart rate (HR) recorded in the ‘steady state’ at walking speed V = 1.25 and 1.75 m^.^s^-1^ were used to determine the most suitable line of HR and VO_2_ for each subject. VO_2_max was then estimated based on the predicted value of maximum heart rate (HRmax) calculated using formula 3 [30]:

$$HR_{max}=208-0.7\times age$$ (3)

For each subject VO_2_max was estimated twice, i.e. based on HR and VO_2_ values recorded in sub-maximum NW effort, and based on the same indices recorded in sub-maximum W effort. VO_2_max was expressed as an absolute value [l^.^min^-1^] or as a value relative to body weight [ml^.^kg^.^min^-1^].

### Statistical methods

The results were analysed using Dell Statistica version 13.1 (StatSoft, Poland). Test results are presented in the form of mean values (M), standard deviation (SD) and 95% confidence intervals. Normal distribution of data was tested using the Shapiro-Wilk test. The influence of training on the level of indices in the case of variables measured only twice (before and after a series of workouts) was analysed using the Student’s t-test for dependent samples. To assess the significance of differences between mean values of the remaining variables the one-way analysis of variance (ANOVA) was used. In the case of rejection of the hypothesis of equality of means, significantly different mean values were determined using the Tukey’s test (HSD). The adopted significance level was α = 0.05 for all tests.

## Results

The 6-week NW training at individually defined intensity resulted in a significant reduction (p ≤ 0.02) of total body mass (BM), fat mass (FM) and body mass index (BMI) in the subjects, without significant changes in lean body mass (LBM) (Table 1).

**Table 1:** Anthropometric characteristic of the study subjects before (b) and after (a) a 6-week Nordic Walking training.

Variable	Parameters of respondents (n = 18)			
	b	a	Δ	p-value*
	M ± SD	M ± SD	M ± SD	
BM (kg)	69.3 ± 9.01	68.7 ± 8.66	0.62 ± 1.00	0.02
LBM (kg)	45.2 ± 4.79	45.0 ± 4.54	0.19 ± 0.77	ns
FM (kg)	24.2 ± 4.83	23.7 ± 4.68	0.43 ± 0.55	<0.01
PBF (%)	34.6 ± 3.14	34.3 ± 3.19	0.26 ± 0.66	ns
BMI (kg^.^m^-2^)	26.6 ± 2.79	26.4 ± 2.70	0.28 ± 0.33	<0.01

Values are mean (M) ± standard deviation (SD); Δ—changes in indicator levels resulting from the workout; BM- body mass; LBM—lean body mass; FM—fat mass; PBF—percent of body fat, BMI—body mass index; ns—differences not statistically significant

* the results of the Student’s t-test for dependent samples.

The mean values of heart rate (HR) and systolic (SBP) and diastolic (DBP) blood pressure values recorded at rest were significantly reduced (p < 0.01) (Table 2).

**Table 2:** Haemodynamic indices at rest before (b) and after (a) a 6-week Nordic Walking training.

Variable	Parameters of respondents (n = 18)			
	b	a	Δ	p-value*
	M ± SD	M ± SD	M ± SD	
HR_rest_ (beats^.^min^-1^)	85.9 ± 14.0	76.6 ± 12.3	9.33 ± 10.5	<0.01
SBP (mm Hg)	127.5 ± 10.3	116.1 ± 11.5	11.4 ± 9.52	<0.01
DBP (mm Hg)	80.3 ± 8.13	74.4 ± 7.05	5.83 ± 6.24	<0.01

Values are mean (M) ± standard deviation (SD); Δ—changes in indicator levels resulting from the workout; HR_rest_−heart rate at rest; SBP–systolic blood pressure; DBP–diastolic blood pressure

* the results of the Student’s t-test for dependent samples.

The training intervention resulted in significant changes in the mean values of circulatory and respiratory indices recorded during the graded walking exercise on a mechanical treadmill with poles and without poles in the segment at walking speed of 1.75 m^.^s^-1^ in the ‘steady state’ (Tables 3 and 4).

**Table 3:** Cardiorespiratory indices in a graded walking exercise with (NW) and without poles (W) in the segment at walking speed of 1.75 m^.^s^-1^ before (b) and after (a) a 6-week Nordic Walking training.

Variable	Parameters of respondents (n = 18)				p-value*
	W_b_	W_a_	NW_b_	NW_a_	
	M ± SD	M ± SD	M ± SD	M ± SD	
VO_2_∙HR^-1^ (ml^.^beats^-1^)	8.45 ± 1.38	9.82 ± 1.68	9.19 ± 1.24	10.54 ± 1.73	<0.01
∑HR (beats^.^min^-1^)	1459.0 ±155.9	1308.9 ± 154.1	1544.0 ± 175.9	1395.3 ± 170.1	<0.01
FR (l^.^min^-1^)	27.7 ± 5.64	28.3 ± 5.03	32.1 ± 4.48	31.1 ± 4.65	0.03
VE (l^.^min^-1^)	34.3 ± 6.36	33.7 ± 5.99	41.9 ± 8.87	41.7 ± 7.77	<0.01
TV (l)	1.27 ± 0.29	1.20 ± 0.16	1.33 ± 0.23	1.36 ± 0.26	ns
VO_2_ (l^.^min^-1^)	1.18 ± 0.18	1.20 ± 0.18	1.41 ± 0.21	1.42 ± 0.22	<0.01
VO_2_ (ml^.^kg^-1.^min^-1^)	16.8 ± 1.81	17.7 ± 2.72	20.4 ± 2.28	20.82 ± 2.98	<0.01
RER	0.88 ± 0.04	0.84 ± 0.05	0.90 ± 0.06	0.85 ± 0.03	<0.01
EE (cal)	5.90 ± 0.93	5.70 ± 0.87	6.93 ± 1.10	6.78 ± 1.07	<0.01

Values are mean (M) ± standard deviation (SD); VO_2_^.^HR^-1^—oxygen pulse; ∑HR—the sum of heart rate till the end of the segment with 1.75m.sek^-1^ walking speed; FR—frequency of respiration; VE—minute ventilation; TV—tidal volume; VO_2_—oxygen uptake per minute; RER—respiratory exchange ratio; EE—energy expenditure; ns—differences not statistically significant

* a one-way analysis of variance (ANOVA).

**Table 4:** Comparisons in pairs of average values of indicators using the Tukey’s post-hoc test (The * symbol means important significant differences).

Variable	Specification					
	W_b_-W_a_	W_b_–NW_b_	W_b_-NW_a_	W_a_–NW_b_	W_a_–NW_a_	NW_b_–NW_a_
HR (beats^.^min^-1^)	*	*		*	*	*
VO_2_∙HR^-1^ (ml^.^beats^-1^)	*		*			*
∑HR (beats^.^min^-1^)	*			*		*
FR (l^.^min^-1^)		*		*		
VE (l^.^min^-1^)		*	*	*	*	
VO_2_ (l^.^min^-1^)		*	*	*	*	
VO_2_ (ml^.^kg^-1.^min^-1^)		*	*	*	*	
RER	*			*		*
EE (cal)		*	*	*	*	
SBP^3ʼ^ (mm Hg)	*		*	*		*
DBP3ʼ (mm Hg)	*		*	*		*
VO_2_ max(l^.^min^-1^)	*		*			*
VO_2_ max(ml^.^kg^-1.^min^-1^)	*		*			*

W–regular walking; NW–Nordic walking; b—before training intervention; a—after training intervention; VO_2_^.^HR^-1^—oxygen pulse; ∑HR—the sum of heart rate till the end of the segment with 1.75m^.^s^-1^ walking speed; FR—frequency of respiration; VE—minute ventilation; TV—tidal volume; VO_2_—oxygen uptake per minute; RER—respiratory exchange ratio; EE—energy expenditure; SBP^3ʼ^—systolic blood pressure in the 3^rd^ minute of restitution; DBP^3ʼ^—diastolic blood pressure in the 3^rd^ minute of restitution; VO_2_max–maximum oxygen uptake.

**Fig 2:**
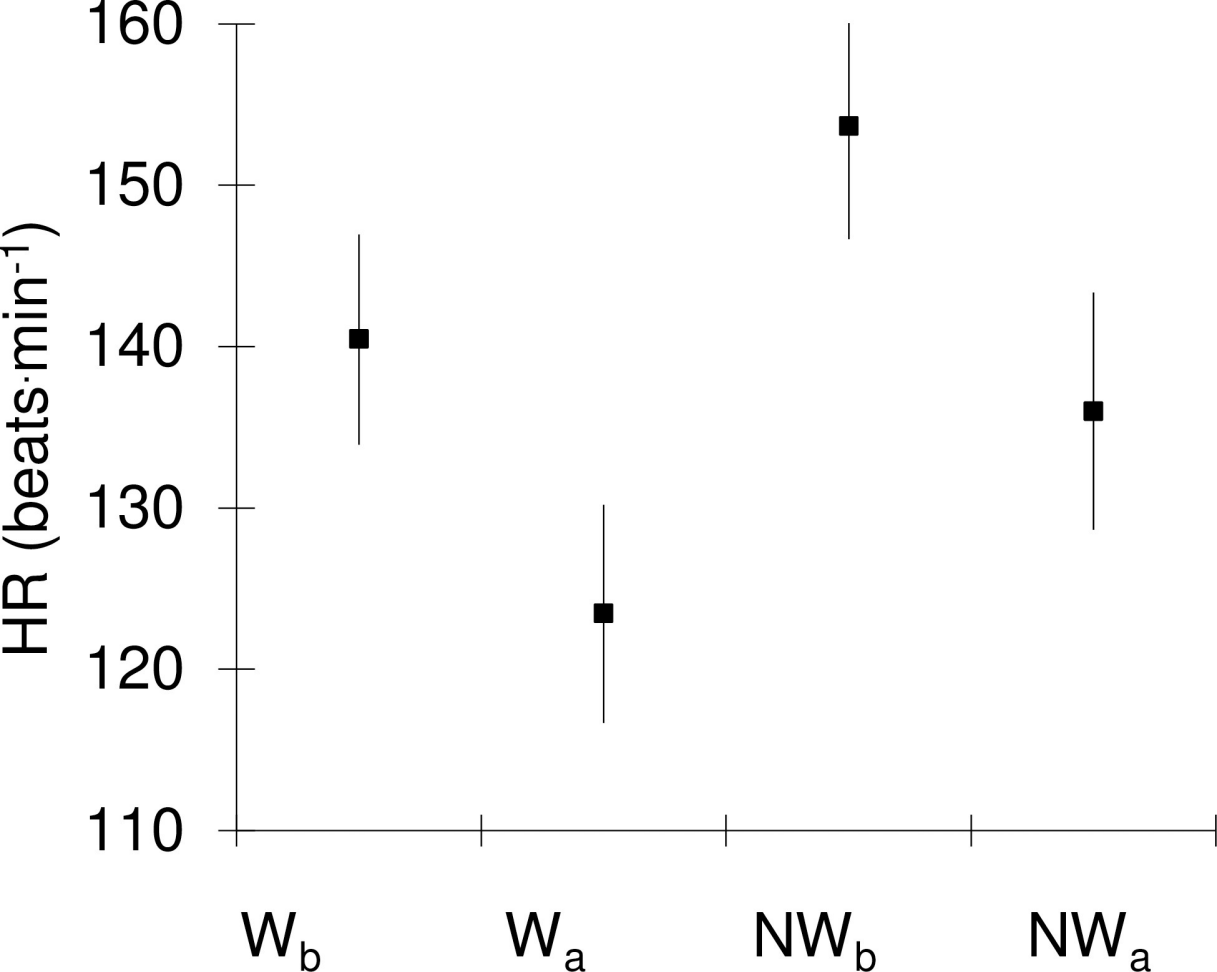
HR in segments at walking speed of 1.75 m^.^s^-1^ before and after NW training. Heart rate (HR) of the study subjects (n = 18) in the graded walking exercise with poles (NW) and without poles (W) before (b) and after (a) a series of workouts. Values are presented as mean and 95% confidence intervals.

At low probability value (p < 0.01) there were significant differences in HR values, which, after completing the training intervention, decreased by 11.5% in NW and by 12.1% in W (Fig 2 and Table 4).

The differences between the average values of oxygen pulse (VO_2_∙HR^-1^) were statistically significant (p < 0.01). The average values in NW increased by 14.7% as a result of training and by 16.2% in W (Tables 3 and 4). The analysis of variance also revealed that the mean results of the sum of heart rate (ΣHR) were significantly different (p < 0.01), so were the RER values recorded in the segment with walking speed of 1.75 m^.^s^-1^ (p < 0.01). As a result of training, the mean value of ΣHR was reduced by 9.6% in NW and by 10.3% in W, while the average RER values decreased in both the NW and W exercises by 5.6% and 4.5% respectively. At the same time, the average values of frequency of respiration (FR), minute ventilation (VE), tidal volume (TV), oxygen consumption (VO_2_) and energy expenditure (EE) indices recorded in this load segment after the training period in both NW and W did differ not significantly from the values obtained in the initial measurements (Tables 3 and 4).

Comparing the physiological response of a female body to walking exercise on a mechanical treadmill showed that the mean values of HR, FR, VE, VO_2_ and EE indices recorded during NW in the segment with walking speed at 1.75 m^.^s^-1^ differed significantly (p <0.05) from the values obtained during W at the same walking speed. In both the baseline measurements and the post-training measurements, the average HR values (Fig 2), V_E_, VO_2,_ and EE during the NW exercise were significantly higher than the values recorded in the same load segment during W. At the same time, the mean FR values were significantly higher in NW in pre-workout measurements (Tables 3 and 4).

The NW training did not significantly affect the rate of HR recovery (HRR%) after NW or after W. The low test probability value (p < 0.01) indicates that the average SBP and DBP values recorded in the 3^rd^ minute after the NW exercise, and in the 3^rd^ minute after the W exercise presented statistically significant decrease in relation to the values recorded in identical measurements before the training intervention (Tables 4 and 5).

**Table 5:** Haemodynamic indices in the 3^rd^ minute of recovery after graded walking exercise with (NW) and without poles (W) before (b) and after (a) a 6-week Nordic Walking training.

Variable	Parameters of respondents (n = 18)				p-value*
	W_b_	W_a_	NW_b_	NW_a_	
	M ± SD	M ± SD	M ± SD	M ± SD	
HR^3ʼ^ (beats^.^min^-1^)	97.9 ± 11.0	90.5 ± 9.95	101.1 ± 9.64	99.0 ± 14.3	ns
HRR%	85.9 ± 13.9	83.5 ± 10.3	81.8 ± 14.9	77.5 ± 11.9	ns
ΔHR_max-3ʼ_ (beats^.^min^-1^)	58.1 ± 10.3	60.3 ± 11.6	58.9 ± 10.7	58.8 ± 9.03	ns
SBP^3ʼ^ (mm Hg)	136.7 ± 11.1	120.8 ± 8.95	135.6 ± 7.65	122.2 ± 9.27	<0.01
DBP^3ʼ^ (mm Hg)	85.3 ± 6.52	76.4 ± 6.37	86.1 ± 6.31	77.8 ± 6.91	<0.01

Values are mean (M) ± standard deviation (SD); HR^3ʼ^—heart rate in the 3rd minute of restitution; HRR%—heart rate recovery index; ΔHR_max-3ʼ_—maximal heart rate during the exercise test minus the heart rate in the 3^rd^ minute of restitution; SBP^3ʼ^—systolic blood pressure in the 3^rd^ minute of restitution, DBP^3ʼ^—diastolic blood pressure in the 3^rd^ minute of restitution; ns—differences not statistically significant

* a one-way analysis of variance (ANOVA).

**Fig 3:**
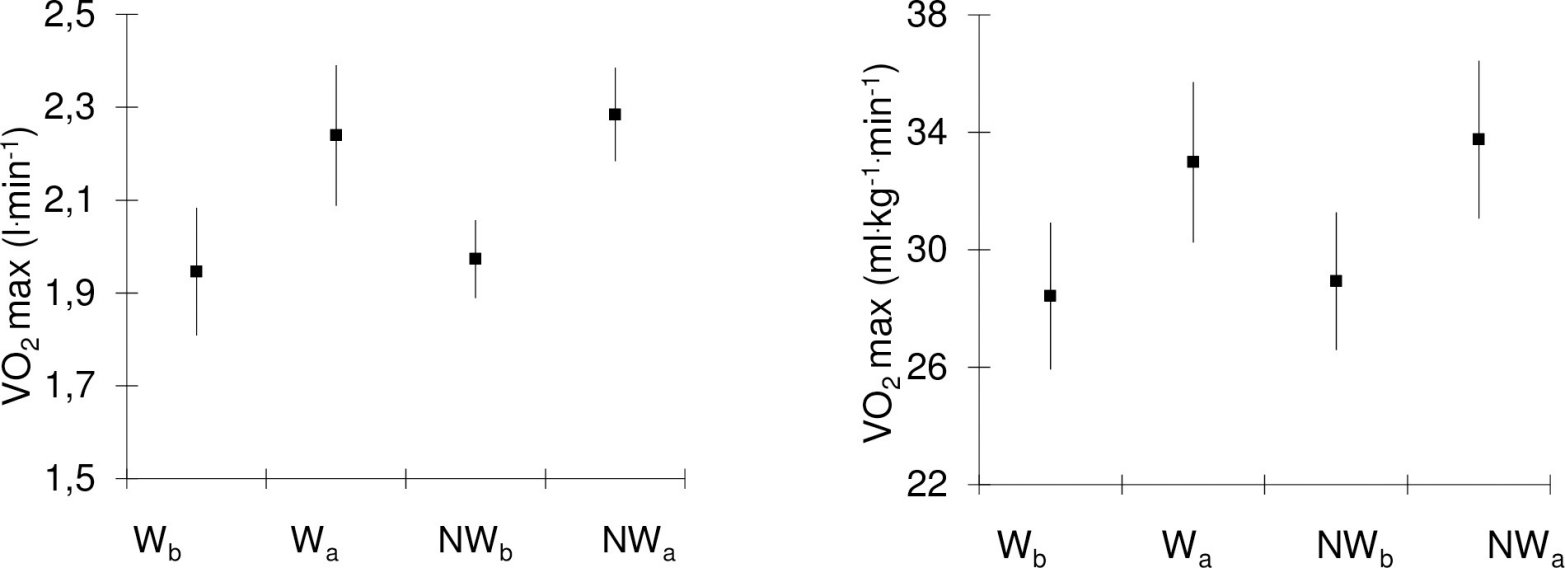
VO_2_max before and after a 6-week Nordic Walking training. Predicted values of maximum oxygen uptake (VO_2_max) of the study subjects (n = 18) based on the sub-maximum walking exercises with poles (NW) and without poles (W). Values are presented as mean and 95% confidence intervals.

After the 6-week training a significant increase in both the absolute (l^.^min^-1^) and the relative (ml^.^kg^.^min^-1^) values of the VO_2_max index was revealed. In baseline measurements, the mean relative value of VO_2_max predicted from the sub-maximum NW effort was 28.93 ± 4.71 ml kg^-1.^min^-1^ and the one based on W effort was 28.42 ± 5.03 ml kg^-1.^min^-1^ (Fig 3). The relative VO_2_max values increased significantly by 16.7% in NW and by 16% in W. Both the absolute and the relative values of VO_2_max predicted from the sub-maximum NW effort did not differ significantly from the values predicted on the basis of the W effort (Fig 3 and Table 4).

## Discussion

The 6-week NW training intervention at the intensity aimed at the dominant mobilisation of fatty reserves resulted in beneficial changes in body composition, manifested by a significant reduction in BM, FM and BMI in the previously sedentary women. These changes were not accompanied by a significant reduction in LBM, which is consistent with the results of similar studies, in which the intensity of exercises in training corresponded with the maximum rate of fat oxidation (Fatmax) or the lowest value of respiratory quotient (RQ) [21,31,22]. Considering age-related changes in body composition, namely progressive reductions in lean body mass and increases in adipose tissue [32–34], such an intensity of NW training in older people seems to be particularly beneficial health-wise [24]. What is interesting, the study conducted by Drapier et al. [35] showed that the effects of LIPOXmax training continue to improve body composition within 3 years. The results of our study suggest that the applied procedure is likely to have this effect. Therefore, long-term studies with similar intensity of NW exercises are justified.

The NW training resulted in a significant reduction of both the resting and the exercise HR values. Also, the decrease of the sum of heart rate reached in the NW graded exercise, similarly to the W exercise, was statistically significant, while oxygen pulse increased. This, proves that was is an improvement of myocardial function, including a more efficient response to increased workload.

The obtained results are consistent with the results of the studies by Wang et al. [22], who recorded reduction in resting HR values after a 10-week Fatmax training programme, which included ordinary walking or jogging, in overweight middle-aged women. As some researchers suggest, endurance training improves the cardiac baroreceptor reflex sensitivity, due to reduced arterial stiffness [36]. In post-menopausal women, such post-training reduction in resting HR values may also be a manifestation of improvement in cardiovagal regulation [37].

Age-related vascular endothelial dysfunction, peripheral vascular resistance, and arterial stiffness may predispose to the development of hypertension [38], which significantly increases the risk of cardiovascular disease and death from any cause [39]. In post-menopausal women, the increased activity of the sympathetic nervous system may be an additional factor predisposing to the development of hypertension, which seems to result from the age-related reduction in baroreceptor reflex sensitivity [40].

In the present study, despite the post-menopausal phase, the average resting BP values recorded in the initial measurements, in the examined women were within the range of values considered normal, although they exceeded the optimal BP level (BP < 120/80) [41]. As a result of the training, both the systolic and diastolic BP values significantly decreased, which is consistent with the results of many previous studies, indicating that aerobic training, including NW, is an effective non-pharmacological method of controlling blood pressure in the elderly [42,43]. These results confirm the hypotensive effect of exercises with intensity aimed at maximum fat oxidation, as reported by Wang et al. [22], and prove the effectiveness of such an intervention even in the normotensive population. Such reduction of BP induced by exercise, according to some researchers, seems to be the result of functional rather than structural adaptation of arterial walls [44], which may counteract the age-related increases in this index and thus reduce the risk of adverse cardiovascular events [45,46].

After the 6-week NW training there was a significant reduction in mean RER values in the segment of walking at speed of 1.75 m^.^s^-1^ both in the NW and W workouts, which is consistent with the results of similar studies [22]. This phenomenon can be explained by the positive impact of endurance training on fat oxidation and the increased ability of mitochondria to oxidise fatty acids. A lower RER value means that with a specific workload a trained body uses more energy from fat deposits during exercise. This post-exercise increase in lipid oxidation during moderate-intensity exercises has already been documented [47].

NW training did not significantly affect the heart rate recovery index after exercise (HRR%), which may suggest that the bodies of the elderly needs more time before the haemodynamic indices return to the resting level. This may be the result of reduced efficiency of the autonomic nervous system, due to aging [48]. It was observed, however, that after the period of training, the blood pressure values (SBP and DBP) recorded in the 3^rd^ minute after the NW and W exercises were significantly lower in comparison with the values obtained after identical effort in the pre-training measurements. Lower BP during recovery recorded after the workout may be associated with the improved aerobic performance of the subjects, already reported by other researchers [49]. It is known that the time of the HR and BP values returning to the resting levels after exercise depends not only on the efficiency of the autonomic nervous system, but also on physical fitness [49]. As anticipated, after NW training, the increase of the values of VO_2_max, predicted by sub-maximum efforts with poles and without poles, was statistically significant. The increase in aerobic performance and the associated greater muscle relaxation contribute to the reduction of blood pressure after exercise, in which the hypotensive effect of exercise can be observed [50].

The improvement of aerobic performance in the elderly as a result of NW training has been also reported by other researchers [6]. However, contrary to the observations of Latosik et al. [43], in the present study a significant increase in VO_2_max was recorded as early as 6 weeks into the training. These results suggest that the correctly selected intensity of exercise may be conducive to faster adaptation of the body to exercise in older women. The improvement of aerobic performance resulting from physical training may have protective implications for the aging circulatory system and the quality of life of the elderly [6].

The present study confirmed the observations of other authors [8,9] indicating that the use of poles during walking increases the intensity of effort, which is associated with higher HR, VE, VO_2,_ and EE in relation to regular walking at the same speed. What is interesting, in this study, the differences in physiological parameters between the NW and W exercises were similar, both before and after 6 weeks of training. In the pre-training measurements, HR during the NW was higher in relation to HR recorded during the W by 9.4% and after the training by 10.2%. EE was also higher in the NW exercise before (by 17%) and after the training (by 18.9%). VE was higher by 22.1% and 23.7%, respectively. On the other hand, the differences in the level of VO_2_ (l^.^min^-1^) after the training slightly decreased in comparison to those recorded in the initial measurements (19.5% vs. 18.3%), which, however, can be explained to some extent by the effect of training.

Considering the observations of some researchers suggesting that the range of differences in the levels of physiological parameters between NW and W may be affected by both the ineffective use of poles during walking on treadmill [51] and the level of NW skills [52], it can be assumed that in this study its subjects sufficiently mastered the technique of walking with poles before starting the training. This resulted in the correct use of poles, when walking on mechanical treadmill. It allowed to reduce the risk of possible misrepresentation of results, due to the incorrect use of poles, and, also, increased the effectiveness of the training programme [53].

This study had its limitations. The relatively small sample size may not have allowed to generalise the obtained results, however, it allowed close supervision by specialists, which helped in maintaining the subjects’ engagement in the training programme. This study had a single group pre-test/post-test design without a control group. In order to fully confirm the superiority of the applied intensity in training for health over the intensity determined in a standard manner, further research is necessary with the presence of a control group.

## Conclusions

The 6-week NW training performed at the intensity corresponding to the dominance of lipid metabolism has improved body composition, the function of the cardiovascular system and physical performance in post-menopausal women over the age of 55. This proves the effectiveness of such an intervention in improving health of the elderly, and suggests that individual selection of exercise intensity may become an important strategy for planning health training programmes for seniors in the future.

The obtained results prove that regardless of the training intervention, NW compared to regular walking at the same speed generated higher energy expenditure, therefore, its use in aerobic training of the elderly should be strongly recommended.

## Supporting information

S1 Dataset General dataset. (XLSX)

S2 Dataset Dataset to Fig 2. (XLSX)

S3 Dataset Dataset to Fig 3. (XLSX)
